# Effect of Nano-Sized γ′ Phase on the Ultrasonic and Mechanical Properties of Ni-Based Superalloy

**DOI:** 10.3390/nano12234162

**Published:** 2022-11-24

**Authors:** Ziqi Jie, Zhaoning Yang, Tao Xu, Chongfeng Sun

**Affiliations:** 1 School of Materials and Chemical Engineering, Xi’an Technological University, Xi’an 710021, China; 2 School of Science, Xi’an University of Posts & Telecommunications, Xi’an 710121, China

**Keywords:** nano-sized γ′ phase, ultrasonic properties, mechanical properties, IN939 superalloy

## Abstract

The effect of the nano-sized γ′ phase on the ultrasonic and mechanical properties of the IN939 superalloy was investigated. The results indicate that the microstructure characteristics of the nano-sized γ′ phase directly affected the ultrasonic longitudinal velocity, the attenuation coefficient, and the mechanical properties. The ultrasonic longitudinal velocity increased with the volume fraction of the γ′ phase, whereas the attenuation coefficient was similar to the fractional change in the γ channel width. The lower fractional change in the γ channel width, in combination with a high volume fraction of the γ′ phase, was conducive to improving the mechanical properties of the superalloy. Additionally, the variation in the ultrasonic properties could reflect the variation in the mechanical properties of the IN939 superalloy, which was beneficial for optimizing the heat treatment process and characterizing the γ′ phase precipitation behavior in a nondestructive manner.

## 1. Introduction

The IN939 superalloy is extensively used in the gas turbine industry due to its superior mechanical properties, and oxidation and corrosion resistance [1,2]. In order to improve gas turbine energy efficiency and lower carbon dioxide emissions, it is necessary to optimize the service performances of superalloys [3]. Extensive research on nickel superalloys has shown that the morphology, size distribution, and volume fraction of nanometer-sized precipitated phases (γ′ and γ″ phase) in the γ matrix are crucial for obtaining the desired service performances [3,4,5,6,7,8,9,10,11]. The γ′ phase is an A_3_B intermetallic phase, in which A is Ni and Co, and B is Al and Ti; it precipitates from the austenitic γ matrix and has a face-centered cubic crystal structure (ordered L1_2_ structure) [3,5,6]. The size distribution and volume fraction of the nanometer-sized precipitated γ′ phase are highly dependent on the temperature and time of the heat treatment [9,10,11]. Therefore, selecting appropriate heat treatment processes is crucial for the service performances of superalloys.

Ultrasonic properties, which result from the interactions between the ultrasonic wave and the microstructures of materials, have been extensively used for optimizing heat treatment processes [12,13] and characterizing the microstructures [14,15] and mechanical properties [16,17] of materials. Ultrasonic velocity and attenuation reflect variations in the grain size [18,19], volume fraction, the size of the precipitated phase [20,21,22,23], the type of precipitated phase [15,24], and the defects during the heat treatment of superalloys. Ultrasonic velocity has been reported to increase with the formation of the precipitate phases, whereas the dissolving or coarsening of precipitates leads to decreases in velocity [12,14,15,16,20,21,22,25]. This is mainly because the formation and dissolution of the intermetallic precipitates change the composition of an alloy, improving the elastic modulus and increasing the ultrasonic velocity [14,16,20,22]. The ultrasonic velocity of an alloy increases with the increase in the volume fraction of the nano-sized γ′ phase and η phase, but carbide has little effect on ultrasonic velocity [14,15,24]. Further, it has been found that the change in ultrasonic velocity caused by the change in the microstructure of a superalloy is consistent with the change in mechanical properties. Additionally, various precipitates scatter sound waves, which influence attenuation values [21,26]. The phase change and microstructure characteristic parameters show strong correlations with variation trends in the ultrasonic attenuation coefficient. During the aging treatment of the IN625 alloy, Laves phase dissolution and Ti- and Nb-rich phase precipitation led to a decrease in the attenuation value [26]. The attenuation coefficient, contrary to the change trend of the ultrasonic velocity, was found to be consistent with the fractional variation in the γ channel width of the superalloy [21]. In conclusion, the aforementioned correlations have shown that ultrasonic velocity and attenuation can reflect the formation, morphologies, and microstructure characteristics of the precipitation phases during the heat treatment of superalloys.

In this study, various microstructure characteristics of the nano-sized γ′ phase in the IN939 superalloy were prepared using different aging treatment processes. To evaluate the nano-sized γ′ phase effect and obtain an optimal heat treatment process, the ultrasonic properties, microhardness, and tensile properties of aging-treated samples were measured. Additionally, the relationships between the ultrasonic properties and mechanical properties of the IN939 superalloy were obtained, which is beneficial for optimizing the heat treatment process and characterizing the γ′ phase precipitation behavior in a nondestructive manner.

## 2. Experimental Procedures

The IN939 superalloy (provided by Jiangsu Longda Superalloy Material Co., Ltd., Wuxi, China) employed in this work had the main chemical composition (wt.%): Cr, 22.21; Co, 18.84; Ti, 3.66; W, 1.96; Al, 1.99; Ta, 1.36; Nb, 1.08; C, 0.15; Zr, 0.06; B, 0.006; and the rest of Ni.

The cylinder samples of Φ30 × 110 mm underwent four different heat treatment processes to produce various microstructures. All samples were treated with solid solution at 1160 °C for 4 h. The aging temperatures of the HT1 and HT2 samples were 850 and 1000 °C, respectively. The HT3 sample was treated with two-step aging, and the HT4 sample was treated with a standard heat treatment process. The sample codes and corresponding heat treatment processes are listed in Table 1.

The microstructures were investigated using scanning electron microscopy (SEM, Helios G4 CX, Thermo Fisher Scientific, Waltham, MA, USA). Moreover, the morphology evolutions and composition of the γ′ phase were also identified using transmission electron microscope (TEM, Talos F200X, Thermo Fisher Scientific, Waltham, MA, USA) and an energy dispersive spectroscopy detector. Image-J 6.5 analysis software was used to examine the microstructure features of γ′ phase after various heat treatments using the same methods as those in reference [27,28,29]. The morphology of the γ′ phase is connected to the measurement of the particle size. The size of a cubic γ′ phase is equal to the average of the length and width, whereas the size of a spherical phase is its diameter.

The ultrasonic properties of cubic samples with dimensions of 20 × 20 × 3 mm were evaluated at ambient temperature following the various aging treatments using a 20 MHz delay line transducer (Olympus company, Waltham, MA, USA) in contact pulse-echo and immersive pulse reflection modes. The ultrasonic longitudinal velocity and attenuation coefficient were calculated using the same methods as those in reference [21].

Vickers hardness testing was conducted using an indentation tester (HMV-G20ST, Shimadzu, Japan) with a 9.8 N load and a 15 s holding time. Ten different sites on each sample were used to measure hardness, and the average values were used to obtain the hardness value. Bar-shaped samples with gauge lengths of 35 mm and diameters of 5 mm were used for tensile testing. The room-temperature tensile properties were tested by universal testing machine (INSTRON 3382, INSTRON, MA, USA) at a consistent strain rate of 2.5 × 10^−4^ s^−1^, and testing was repeated three times to verify data accuracy.

## 3. Results and Discussion

### 3.1. Microstructural Evolution

**Figure 1:**
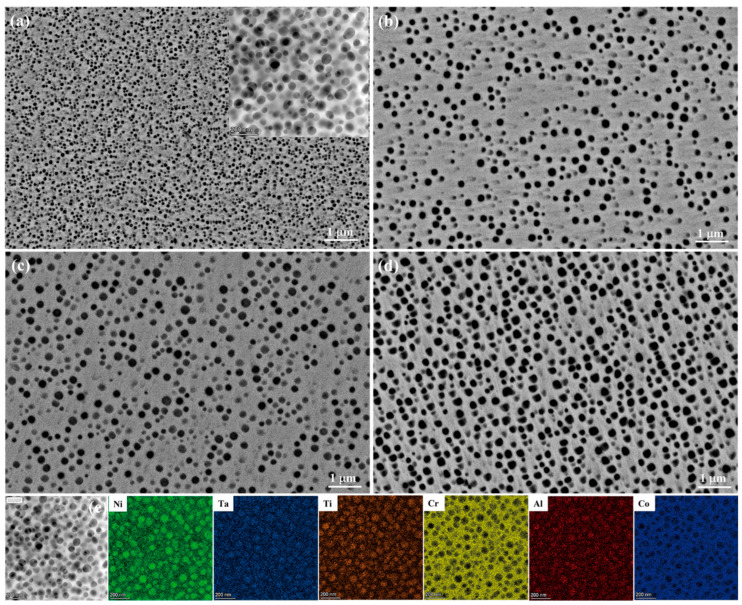
The morphology evolutions of γ′ precipitates for samples (**a**) HT1, (**b**) HT2, (**c**) HT3, and (**d**) HT4, and (**e**) HAADF-TEM image and the EDS map of the γ′ phases in the HT1 sample.

Figure 1 illustrates the morphology evolutions of the γ′ phase in the IN939 superalloy samples under various heat treatment conditions. The energy spectrum analysis (EDS) of the TEM shows that the γ′ phases were mainly in the form of (Ni, Co)_3_ (Al, Cr, Ta, Ti) (Figure 1e). In addition, the aging treatments showed a substantial impact on the morphology of the γ′ phase, which changed its shape from spherical to cuboid. It is evident from the SEM image and the inserted HAADF-TEM image in Figure 1a,b that the morphology of the γ′ phase in the HT1 and HT2 samples is spherical. The HT3 and HT4 samples contained spherical and near-cuboid γ′ precipitates in comparison to the HT1 and HT2 heat-treated samples. The morphological changes of the γ′ particles are mainly due to the competition between elastic strain energy and interface energy under various aging conditions [3,7,30,31]. Due to the fine size of the γ′ phase, the lattice mismatch between the γ matrix and γ′ phase is generally low for the HT1 and HT2 samples. As a result, the isotropic interfacial energy causes the spherical γ′ phase to form. With further coarsening and undergoing Ostwald ripening of the γ′ phase, the elastic strain energy increases faster than the interfacial energy [5,7,30,31]. At this time, the elastic strain energy controls and predominates the spherical to the cuboidal shape of γ′ in the HT3 and HT4 samples.

**Figure 2:**
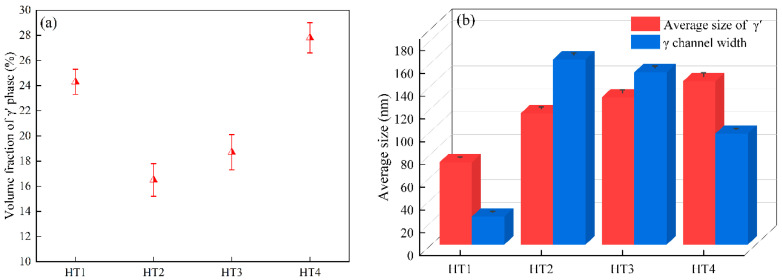
(**a**) The variations in volume fraction of the γ′ phase; (**b**) the variations in γ channel width and γ′ precipitate size under various aging treatment conditions.

Figure 2 depicts the volume fractions and sizes of the γ′ phase and γ channel width under various aging treatments. From the TEM micrographs considering over 200 precipitates, the size of the γ′ phase in the HT1 sample was the smallest (72 nm), and the volume fraction was 24.3%. However, compared with the HT1 heat treatment, the sizes of γ′ precipitated from the HT2 sample (115 nm in diameter) and HT3 sample (129 nm in length) were significantly increased, whereas the volume fractions were decreased. For the standard four-step heat treatment, the average size of the γ′ phase was approximately 143 nm in length, and the volume fraction was 27.8% in the HT4 sample. In addition, the variations in the γ channel width obtained through the image analysis are also displayed in Figure 2b. The narrowest γ channel width was found in the HT1 sample, whereas the widest was found in the HT2 sample. The γ′ phase grows with the core of small γ′ phase particles precipitated by the solid solution process. Its growth is controlled by the diffusion of the alloying elements caused by aging temperature and time. With the increase in aging temperature and time, the faster the diffusion rate of alloying elements and the faster the growth rate of the γ′ phase. Additionally, the Ostwald ripening phenomenon and the merger of the γ′ phase appeared with an increase in the aging temperature and time, which caused the γ′ particle size to gradually grow [3,5,30]. For the HT1 sample, the alloying elements diffuse slowly at low temperatures, increasing the matrix supersaturation, which decreases the critical nucleation energy of the γ′ phase, promotes the nucleation rate, and increases the volume fraction of the γ′ phase. Meanwhile, the short diffusion time prevents the growth of the γ′ phase, resulting in the smaller size of the γ′ phase. In addition, as the aging time is further prolonged, the γ′ phase dissolves and merges, enlarging the matrix γ channel.

### 3.2. Ultrasonic Properties

**Figure 3:**
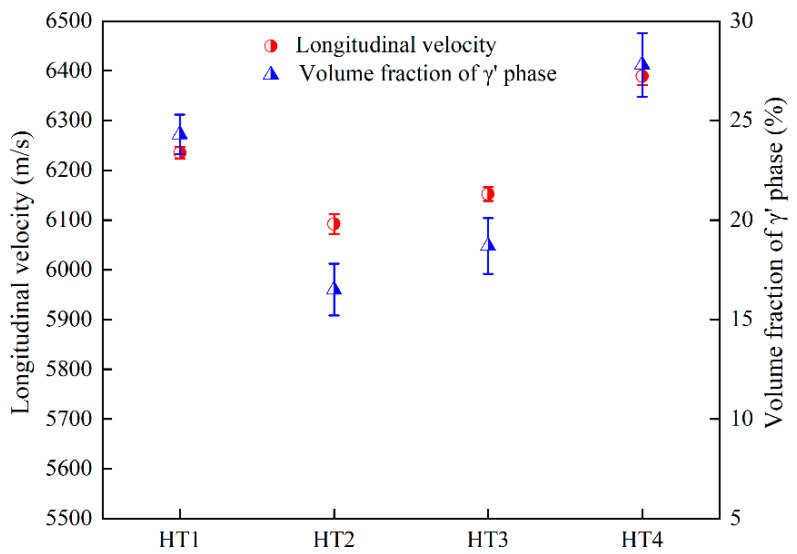
The variation trends in ultrasonic longitudinal velocity and volume fraction of the γ′ phase under various aging treatment conditions.

The effect of the γ′ phase on the ultrasonic longitudinal velocity is shown in Figure 3. The ultrasonic longitudinal velocity increased as the volume fraction of the γ′ phase increased, which is in agreement with previous studies [14,20,21,22]. The minimum ultrasonic longitudinal velocity appeared in the HT2 heat-treated sample, and the corresponding volume fraction of the γ′ phase in the microstructure was 16.5%. However, for the HT4 sample with a 27.8% volume fraction of the γ′ phase, the ultrasonic longitudinal velocity was the largest.

The ultrasonic longitudinal velocity of the alloy was related to both Young’s modulus and density, which is in accordance with the V∝ (E/ρ)^0.5^ equation [14,32]. Therefore, Young’s modulus and density were the major reasons for the variation in the ultrasonic longitudinal velocity of the alloy. The γ′ precipitates throughout the heat treatment process altered the elastic modulus but showed little impact on the density of the alloy. According to a previous study [15,32], the Young’s modulus of the γ′ phase (210 GPa) was approximately 11% higher than the γ matrix (190 GPa). As a result, the Young’s modulus of the superalloy increased with the volume faction of the γ′ phase in the microstructure, which increased the ultrasonic longitudinal velocity of the superalloy. As shown in Figure 3, for the HT4 sample with a 27.8% volume fraction of the γ′ phase, the ultrasonic longitudinal velocity reached the maximum value.

**Figure 4:**
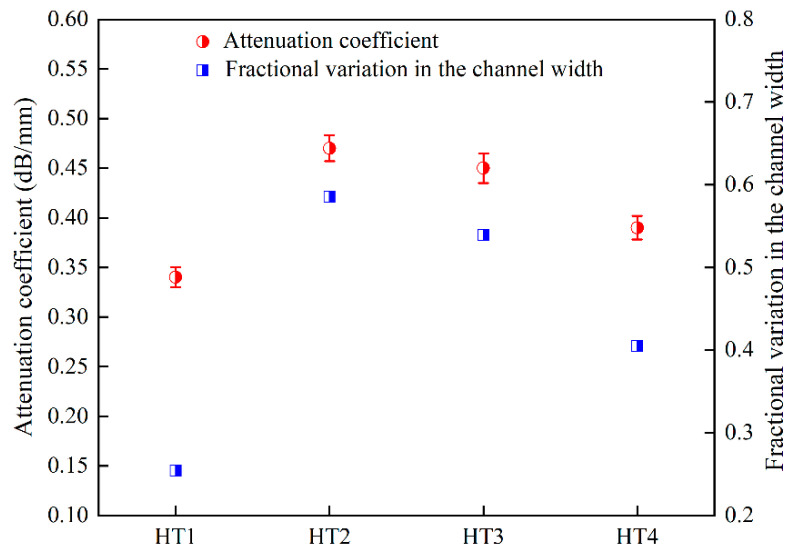
The variation trends in ultrasonic attenuation coefficient and fractional change in the γ channel width under various aging treatment conditions.

Figure 4 indicates the variation trend of the ultrasonic attenuation coefficient of the IN939 alloy after different heat treatment processes. The ultrasonic attenuation coefficient reached the minimum value (0.34 dB/mm) in the HT1 sample and the maximum value in the HT2 sample. For the HT3 and HT4 samples, the attenuation values were close to 0.47 dB/mm, although these two samples showed very different volume fractions and sizes of the γ′ phase. Moreover, it can be seen that the variations in the ultrasonic longitudinal velocity and attenuation presented opposite trends, which is similar to the results of previous research [21]. Mukhopadhyay et al. [21] claimed that the variation trend of the attenuation coefficient induced by variation in the γ′ phase showed a similar trend to the fractional change in the γ channel width (the ratio of the γ channel width to (γ channel width + γ′ precipitation size)), which was primarily governed by a dislocation–damping mechanism. The microstructure analysis in Figure 2 shows that the size of the γ′ phase and the width of the γ channel were affected by different aging treatments. The fractional change in the γ channel width of samples with different heat treatments from small to large was in the order of HT1, HT4, HT3, and HT2, as shown in Figure 4. Therefore, the variation in the attenuation coefficient was mainly due to the fractional change in the channel width, which changed the motion of dislocation in the γ channel during ultrasonic propagation.

### 3.3. Mechanical Properties

**Figure 5:**
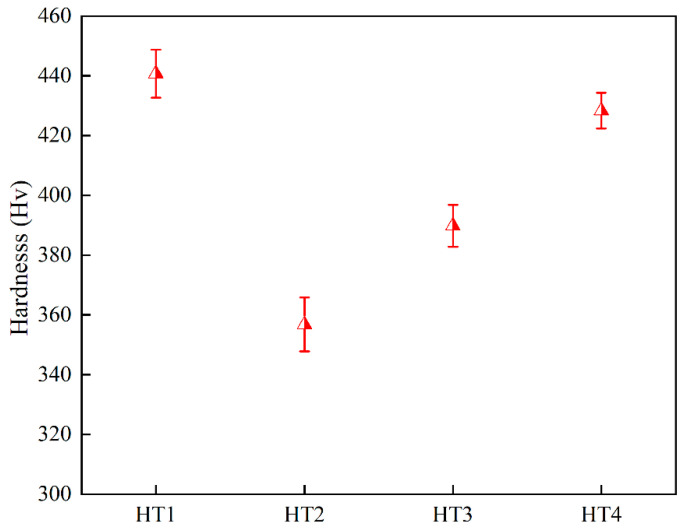
Vickers hardness under different aging treatment processes.

The average Vickers hardness values of the samples under various aging treatments are shown in Figure 5. The hardness of the HT1 sample after low-temperature aging treatment (440 Hv) was significantly higher than the HT2 sample (356 Hv) with high-temperature aging treatment. Moreover, the hardness of the HT3 sample after three-step heat treatment was 9.2% higher than the HT2 sample. After four steps of heat treatment, the hardness of the HT4 sample was close to that of the HT1 sample and 20.2% higher than the HT2 sample.

**Figure 6:**
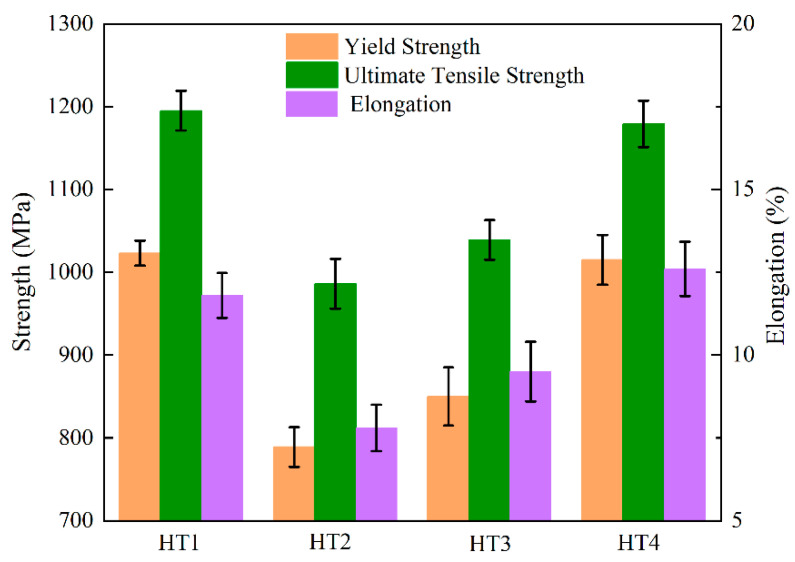
The room-temperature tensile properties under different aging treatment processes.

Figure 6 displays the tensile properties at room temperature for each of the four heat treatment conditions. It is abundantly clear that the γ′ phase significantly affected the tensile properties. The HT1 sample showed a yield strength of 1023 MPa, an ultimate tensile strength of 1195 MPa, and an elongation of 11.8% at room temperature, which is an excellent combination of mechanical properties. However, the HT2 sample, with the lowest volume fraction and largest size of the γ′ phase, showed the lowest tensile properties at room temperature. The HT3 sample showed better tensile properties than the HT2 sample. Moreover, compared with the HT1 sample, the ultimate tensile strength and yield strength decreased slightly, but the elongation increased. These results indicate that the alloy could obtain outstanding room-temperature tensile properties by aging at a low temperature for an extended period of time. A finer size with a high volume fraction of the γ′ phase and a narrower channel of the γ matrix made deformation of the microstructure more difficult, thus increasing the hardness and strength and slightly reducing the ductility [9,10]. According to the results in Figure 1 and Figure 2, high hardness and tensile properties were obtained in the HT1 sample due to the high fraction and dense distribution of the nano-sized γ′ phase.

**Figure 7:**
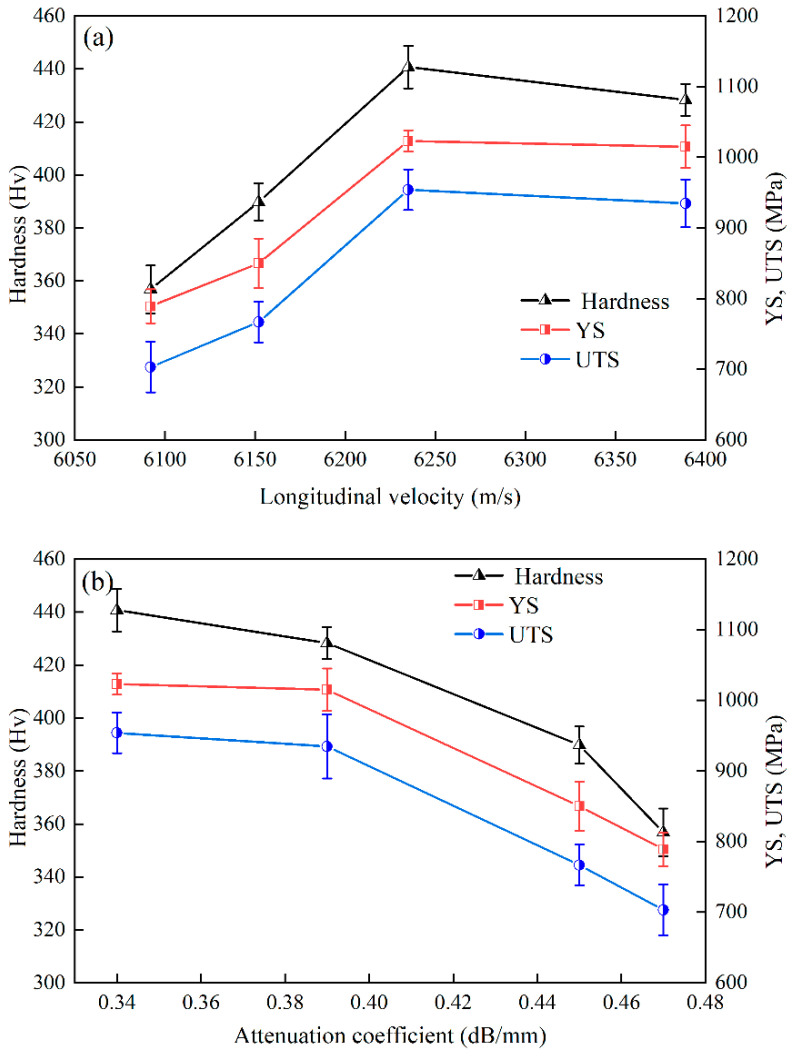
(**a**) Correlation between ultrasonic longitudinal velocity and mechanical properties; (**b**) correlation between attenuation coefficient and mechanical properties.

Figure 7 shows the relationships between the mechanical properties and ultrasonic properties. The results show that the variation in ultrasonic longitudinal velocity was essentially commensurate with variations in hardness and tensile properties in the IN939 alloy, which is consistent with previous research on other nickel-based superalloys [12,22,23]. The longitudinal ultrasonic velocity depended on the change in the elastic modulus caused by the precipitation of the nano-sized γ′ phase in the superalloy. Conversely, the variation trend of the ultrasonic attenuation coefficient was opposite to those of the hardness and tensile properties of the alloy. The mechanical properties of the alloy were mainly affected by the influences of the volume fraction and the size characteristics of the γ′ phase on the dislocation movement. The ultrasonic longitudinal velocity reflected the change in the volume fraction of the γ′ phase, and the attenuation coefficient effectively reflected the size characteristics of the γ′ phase. Therefore, the measurement of the ultrasonic longitudinal velocity and attenuation coefficient could be effectively used to monitor the microstructure variations in superalloys that, in turn, drive changes in the mechanical properties. It could be used as a nondestructive method to characterize microstructure changes in superalloys during heat treatment. According to the results of the relationships between microstructures, and ultrasonic and mechanical properties, the optimal heat treatment process in this paper, considering economy and convenience, was the HT1 process.

## 4. Conclusions

In this paper, the effect of the nano-sized γ′ phase on the ultrasonic and mechanical properties of the IN939 superalloy was investigated.

The aging treatment used showed an important effect on tailoring the morphology and size distribution of the nano-sized γ′ phase. After aging at 850 °C for 24 h, the IN939 superalloy showed less fractional change in the γ channel width, a 24.3% volume fraction of the γ′ phase, and excellent mechanical properties.

The ultrasonic velocity increased with the volume fraction of the γ′ phase, whereas the ultrasonic attenuation and velocity change exhibited opposite trends that were consistent with the fractional change in the channel width.

Additionally, the variation in the ultrasonic properties could reflect the variations in the microstructure and mechanical properties of the IN939 superalloy, which were beneficial for optimizing the heat treatment process and characterizing the γ′ phase precipitation behavior.

## Figures and Tables

**Table 1:** Heat treatment processes for IN939 superalloy.

Sample Code	Heat Treatment Process
HT1	1160 °C/4 h, FAC ^1^ + 850 °C/24 h, AC ^2^
HT2	1160 °C/4 h, FAC + 1000 °C/6 h, AC
HT3	1160 °C/4 h, FAC + 1000 °C/6 h, AC + 800 °C/4 h, AC
HT4	1160 °C/4 h, FAC + 1000 °C/6 h, FAC + 900 °C/24 h, AC + 700 °C/16 h, AC

^1^ FAC: fast air cooling, ^2^ AC: air cooling.

## Data Availability

The data presented in this work are available on request from the corresponding author.
